# Minimally invasive drainage versus open surgical debridement in SAP/SMAP – a network meta-analysis

**DOI:** 10.1186/s12876-019-1078-x

**Published:** 2019-10-21

**Authors:** Kai Zhang, Xiaole Zhu, Chaoqun Hou, Chenyuan Shi, Yi Miao, Qiang Li

**Affiliations:** 1 Pancreatic Center & Department of General Surgery, The First Affliated Hospital of Nanjing Medical University, Nanjing, 210029 Jiangsu China; 2 Pancreas Institute of Nanjing Medical University, Nanjing, 210029 Jiangsu China

**Keywords:** Investigations, Organ failure, Complications, Network meta-analysis

## Abstract

**Background:**

The efficacy of some therapeutic methods (open surgical debridement (OSD), conservative treatment (CST) and minimally invasive drainage (MID)) for severe acute pancreatitis (SAP) and moderately severe acute pancreatitis (MSAP) has been widely evaluated. However, the results remained controversial. We performed this study to illuminate whether any difference in incidence exists on patients with SAP/MSAP treated with OSD and MID.

**Methods:**

Eligible articles were collected base of a comprehensive review of PUBMED, EMBASE, COCHRANE, CKNI and WANGFANG for published randomized controlled trials. Two steps of meta-analysis were performed, routine pair-wise meta-analysis and network meta-analysis.

**Results:**

Thirteen studies were included in this study. Participants were classed as 5 groups, CST, early MID (EMID), late MID (LMID), early OSD (EOSD) and late OSD (LOSD). And MID contains endoscopic drainage (ESD), percutaneous catheter drainage (PCD) and minimally invasive surgery (MIS). Compared with CST, MID could decrease both mortality and multiple organ dysfunction syndrome (MODS) rate but OSD couldn’t. Both EMID and MID can significantly decrease the mortality and MODS rate compared to CST. PCD might be most likely to have a benefit compared to CST.

**Conclusion:**

Existing evidence for the use of MID in SAP/MSAP is reliable and it can be used as early treatment. OSD, if necessary, should be avoided or delayed as long as possible.

**Electronic supplementary material:**

The online version of this article (10.1186/s12876-019-1078-x) contains supplementary material, which is available to authorized users.

## Introduction

Acute pancreatitis (AP) is one of the most common digestive system diseases requiring acute hospitalization worldwide [1]. In Atlanta Criteria revision of 2012 [2], AP was classified based on severity as mild, moderately severe, or severe. Most patients have mild acute pancreatitis and recover without intervention [3–5]. Moderately severe acute pancreatitis (MSAP), which is characterized by local complications in the absence of persistent organ failure, while severe acute pancreatitis (SAP) is defined as persistent single or multiorgan failure (lasting> 48 h) and is present in around 20% of patients, with a mortality rate of 10 to 40% [6, 7]. SAP is a sudden occurrence and an irreversible condition. It usually contains two phases. Early or toxic enzymatic phases in first 2 weeks and later on septic phase after third to fourth week onwards. It occurs with about 20–30% in clinic practices and mortality rate of up to 10–35%. Intervention is generally required for patients with SAP. Timely and effective drainage of abdominal cavity effusion, removal of necrotic tissue and infection control can obviously improve prognosis of SAP include the rate of multiple organ dysfunction syndrome (MODS), complications and death, even the hospitalization time [8]. It is one of the most challenging medical conditions in acute abdomen surgery [9]. In a prospective study, Buchler et al. suggested that patients with infected necrosis remain a high-risk group of SAP, and recommended surgical treatment [10]. Although the open surgical debridement (OSD) was the traditional treatment, it has been associated with high complications and mortality of patients [11, 12]. Many studies have been showed that minimally invasive drainage (MID) may be successfully and safely applied to treat SAP [13]. There is a lack of large-scale randomized clinical trials in this field to compare the effect of MID and OSD [14]. The PANTER trial was the first time to show the feasibility and success of the step-up approach in comparison with open necrosectomy in a randomized controlled manner in the management of SAP [15]. However, it is still bothering us that subset of patients treated by the step-up approach would require laparotomy. Moreover, the timing of surgery is difficult to choose, which remains a problem that needs to be addressed. Therefore, in this review, we mainly aimed to conduct a random-effects routine pairwise meta-analysis and network meta-analysis comparing the safety and efficacy of MID and OSD.

## Methods

### IRB approval and informed consent

This study is a meta-analysis of randomized controlled trials and the IRB approval and informed consent is unnecessary.

### Types of participants

Patients with SAP/MSAP undergoing treatment of CST, MID or OSD were included.

### Types of interventions

We included trials comparing MID (Minimally invasive drainage is small incision surgery and video-assisted surgery. It contains ESD, PCD and MIS), OSD (Open surgical debridement consisted of a laparotomy through an abdominal or retroperitoneal route incision. After removal of all necrotic tissue, drains for postoperative lavage were inserted. And then, the abdomen was closed) and CST (Conservative treatment is an intensive care treatment, which consists of drug therapy (including spasmolysis, analgesia, protease inhibitors), circulatory volume maintenance to prevent electrolyte imbalances, oxygen supplementation, nutritional supplementation, mechanical ventilation). ESD is defined that ERCP is given and nasal biliary drainage or stenting of the pancreatic duct and stone removal were performed under endoscope as appropriate. PCD is a technique that ultrasound-guided percutaneous drainage tube placement. MIS usually means a surgery performed by laparoscope, which make a small incision in the abdomen. Patients whose intervention were started as MID and later converted to OSD belong in MID group. We excluded trials that did not use the above definition. For the operation time, we define that treatments do immediately or early reflected in the studies as early, and those treated late or timing not clear in these studies were categorized as late.

### Types of studies

We only included randomized clinical trials irrespective of blinding or publication status. No language restrictions were used.

### Types of outcome measure

1. Primary outcomes: Mortality.
2. Secondary outcomes: MODS Rate. MODS is thepresence of altered organ function which cannot bemaintained without intervention. It usually involves twoor more organ systems.

### Procedures

We searched the electronic databases up to Aug 2017. We used to search terms included “negative pressure wound therapy”, “negative pressure wound therapies”, “drain”, “drains”, “drainage”, “severe pancreatitis”, “severe acute pancreatitis”, “severe necrotized pancreatitis”, “moderately severe pancreatitis”, “moderately severe acute pancreatitis”, “moderately AND severe necrotizing pancreatitis”, “random allocation”, “random”, “randomly”, “randomized”, “longitudinal studies”. These terms were combined using “AND” and “OR”. References from the acquired articles were also hand-searched. Studies, which were RCT studies for treatment of SAP/MSAP, were included if they met the following criteria: they addressed any types of interventions above and were published in the years 2000–2017. Studies were excluded if they contained any of the following: they were not in English or Chinese, not peer reviewed, and if the main focus did not relate to interventions in SAP/MSAP above. Our search strategy, selection criteria and data extraction were completed independently by two reviewers (Kai Zhang and Xiaole Zhu). We assessed risk of bias in contributing studies with standard criteria [14].

### Study design

We did a network meta-analysis using a Bayesian model. Network meta-analysis can integrate all the data from direct comparisons of treatments within trials and from indirect comparisons of interventions assessed against a common comparator in different trials, to compare all investigated treatments.

### Statistical analysis

**Fig. 1:**
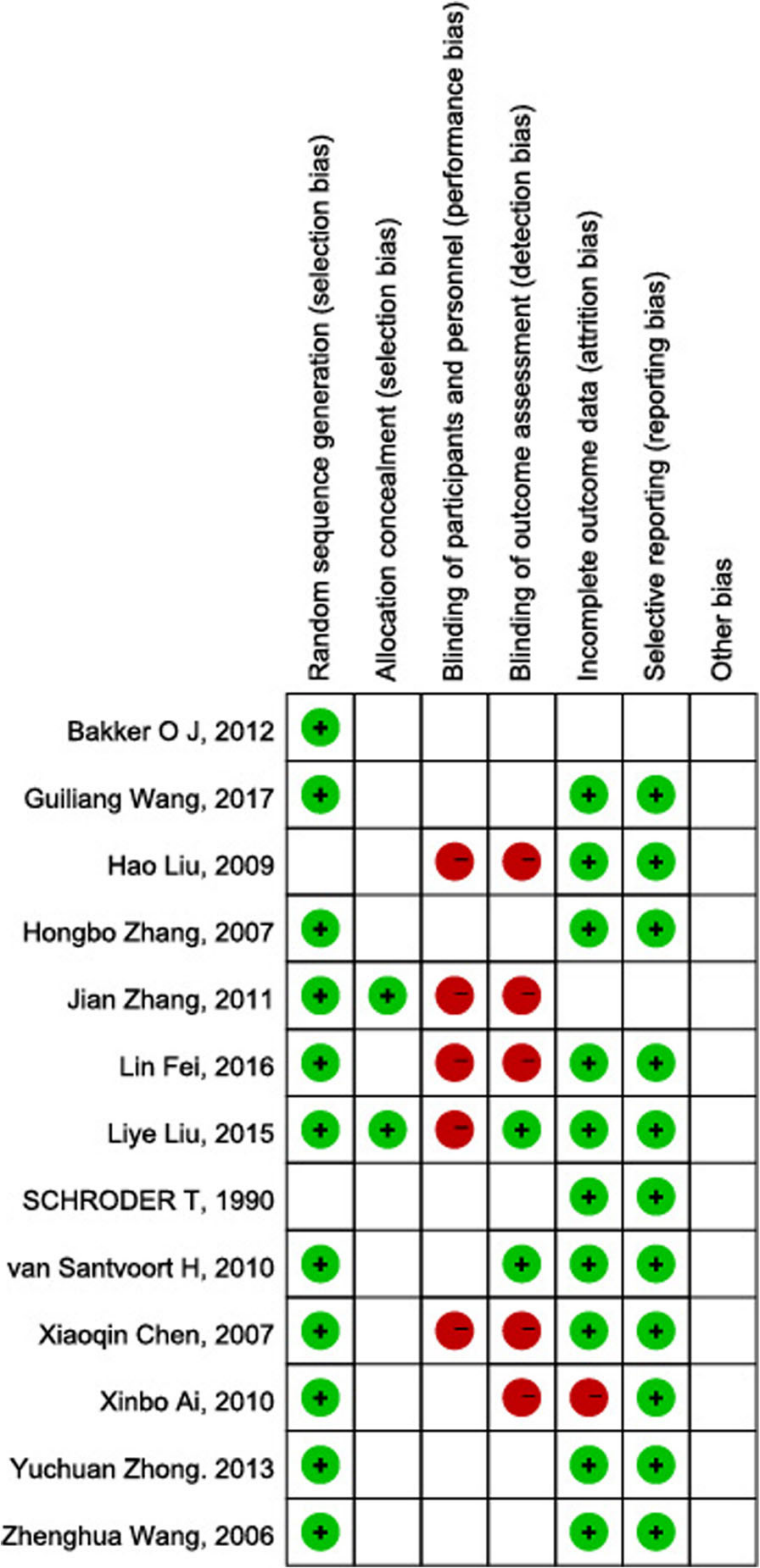
Risk of bias summary

Analysis was performed using RevMan 5.3 (freeware from The Cochrane Collaboration) and R-3.4.2 software. The *gemtc* packages (version 0.8) were used to conduct a Bayesian analysis that combined data from multiple randomized control trials. We assessed the risk of bias of studies in accordance with the Cochrane Handbook for Systematic Reviews of Interventions using RevMan 5.3. The figure of risk of bias summary was showed as Fig. 1. Network meta-analyses are better at integrating different types of evidence compared with conventional pairwise meta-analyses, however this type of analysis leads to inevitable heterogeneity. We did two steps of meta-analysis. First, we did routine pairwise meta-analysis with a random-effects model [17] and I^2^ metric was used to assess the heterogeneity in these analyses [16]. Second, we did random-effects network meta-analysis [18] and the I^2^ metric and meta-regression were also used to assume a common heterogeneity variable for these comparisons by *meta* packages (version 4.9–6). We did routine random-effects pair-wise and network meta-analyses to estimate at primary and secondary outcomes, and we presented the estimates as risk ratios (dichotomous outcomes) with 95% CIs and Crls [19]. The Node-splitting analysis method was used to assess the inconsistency between direct and indirect comparisons when a loop connecting three arms existed. Transitivity is a feature of Network meta-analysis. CST was used as a link to know the relationship between MID and PSD better [20]. Participants who fulfilled our inclusion criteria could be allocated to one of the treatments being compared. Transitivity proceeds if all direct comparisons between treatments respecting to the distribution of effect modifiers (for example, studies comparing MID with CST were similar to studies comparing OSD with CST in terms of the level of mortality).

## Result

**Fig. 2:**
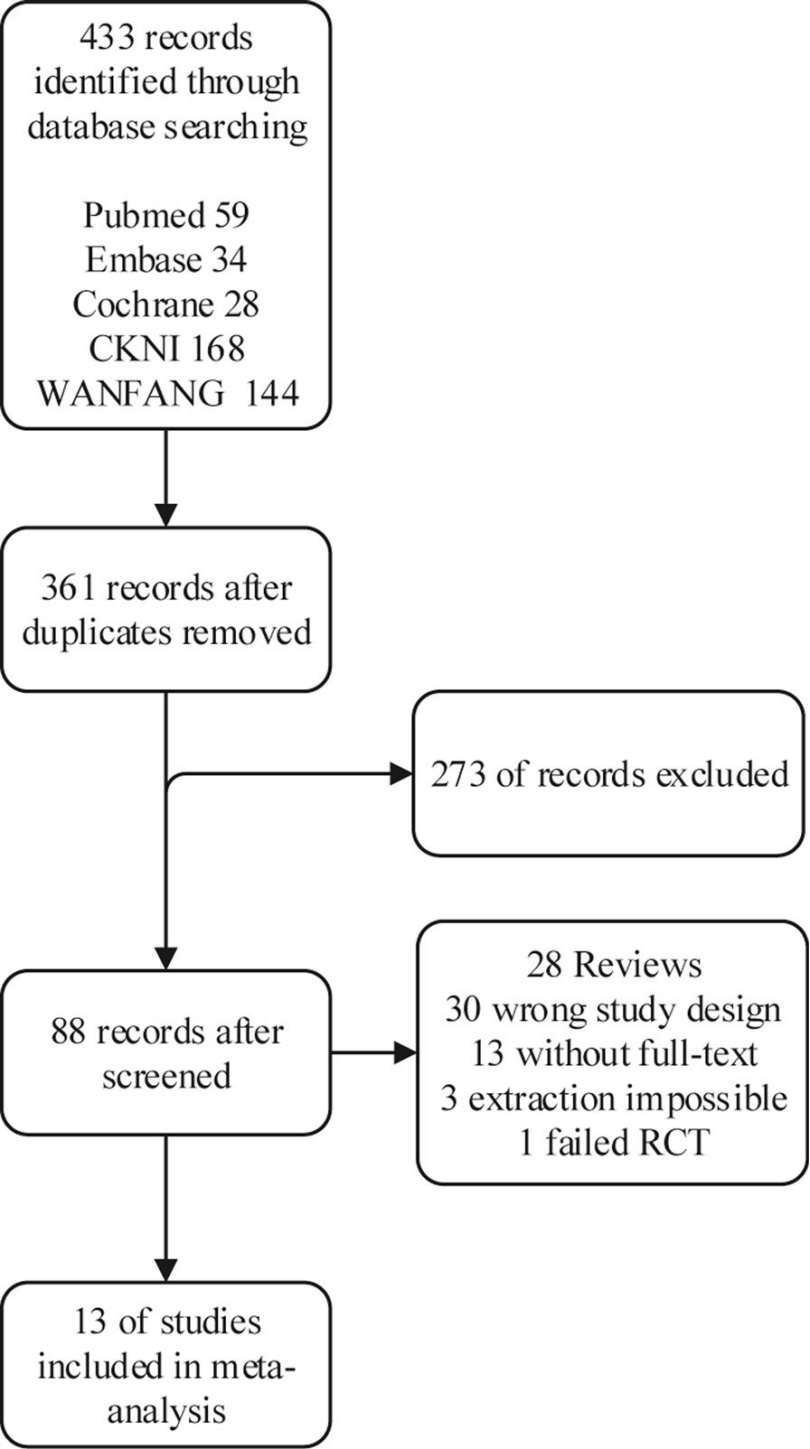
The PRISMA flowchart

**Fig. 3:**
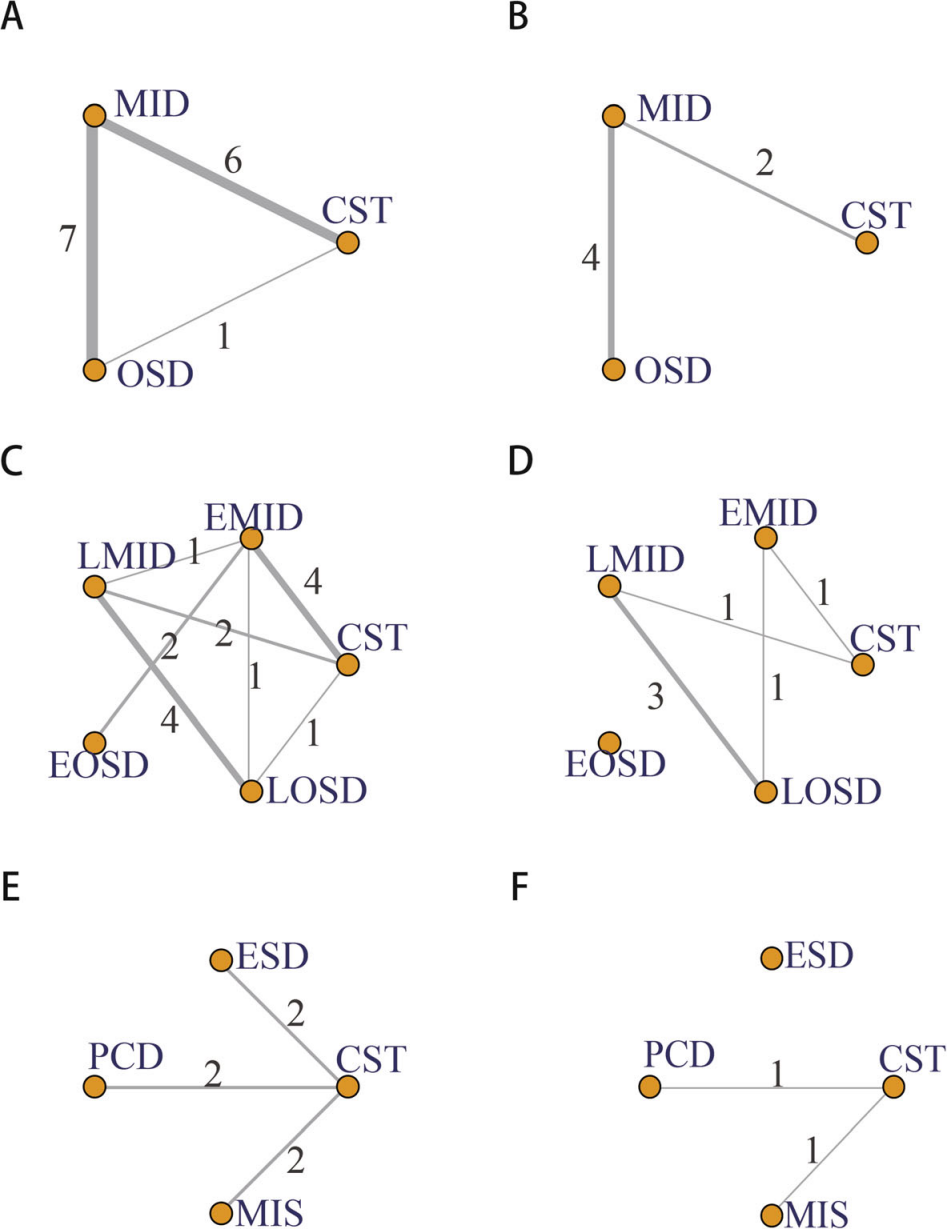
Network of all these comparisons. **a** and **b** for Comparison 1, **c** and **d** for Comparison 2 and **e** and **f** for Comparison 3. The circle stands for one type of therapy, such as MID, CST and OSD. The thickness of lines stand for the number of studies between the two circles (types of therapy) and thicker line means more studies

Finally, thirteen studies with a total of 985 participants were included, which provided enough data for the analyses of mortality conducted in this study. In all of the studies, ten studies were from China while three were from Holland or Finland. Six studies were published in English and the rest were published in Chinese (Table 1). Twelve studies with data for 931 participants were available for the comparison between OSD, MID and CST. Thirteen studies including 985 adults were eligible for the network meta-analysis of different timing for OSD and MID. Six studies with 675 participants were available for the network meta-analysis of different types of MID. The PRISMA flowchart [18] showing electronic searching processes is shown in the Fig. 2. Participants were classed as 5 groups, CST, early MID, MID, early PSD and PSD. And MID contains ESD, PCD and MIS and the comparison between each other was shown in Fig. 3.

**Table 1:** Study characteristics

Author	Year	Country	Language	Study design^a^	Type of intervention	Intervention time	Number of patients	Number of deaths	Number of MODS^b^	Involving groups^c^
Xinbo Ai [29]	2010	China	English	RCT, SC	OSD	late	16	5	3	①②
					MID	late	13	0	1	
Xiaoqin Chen [30]	2007	China	Chinese	RCT, SC	CST	–	12	3	NA	①②③
					OSD	late	14	2	NA	
					MID (ESD)	late	15	2	NA	
Fei Lin [31]	2016	China	Chinese	RCT, SC	MID (ESD)	early	50	1	NA	①②③
					CST	–	50	5	NA	
Van Santvoort HC [27]	2010	Holland	English	RCT, MC	OSD	late	45	7	18	①②
					MID	early	43	8	5	
Hao Liu [32]	2009	China	Chinese	RCT, SC	MID (MIS)	early	35	4	NA	①②③
					CST	–	32	10	NA	
Liye Liu [33]	2015	China	English	RCT, SC	MID (PCD)	early	126	8	NA	①②③
					CST	–	129	11	NA	
Bakker O J [19]	2012	Holland	English	RCT, MC	OSD	late	10	4	5	①②
					MID	late	10	1	0	
Schröder T [34]	1990	Finland	English	RCT, SC	OSD	early	11	3	NA	①②
					MID	early	10	1	NA	
Guiliang Wang [35]	2017	China	English	RCT, SC	CST	–	60	12	11	①②③
					MID (MIS)	early	62	7	7	
Hongbo Zhang [36]	2007	China	Chinese	RCT, SC	MID (PCD)	late	45	0	0	①②③
					CST	–	59	9	6	
Jian Zhang [37]	2011	China	Chinese	RCT, SC	OSD	late	20	6	7	①②
					MID	late	23	1	2	
Zhenghua Wang [38]	2006	China	Chinese	RCT, SC	MID	early	21	1	NA	①②
					OSD	early	20	2	NA	
Yuchuan Zhong [39]	2013	China	Chinese	RCT, SC	MID	early	24	2	NA	②
					MID	late	30	4	NA	

^a^ *RCT* Randomized Controlled Trial, *SC* Single Center, *MC* Multiple Centers

^b^ *NA* Not Available

^C^ ① Comparison 1, ② Comparison 2, ③ Comparison 3

### Comparison 1-MID VS. OSD

**Fig. 4:**
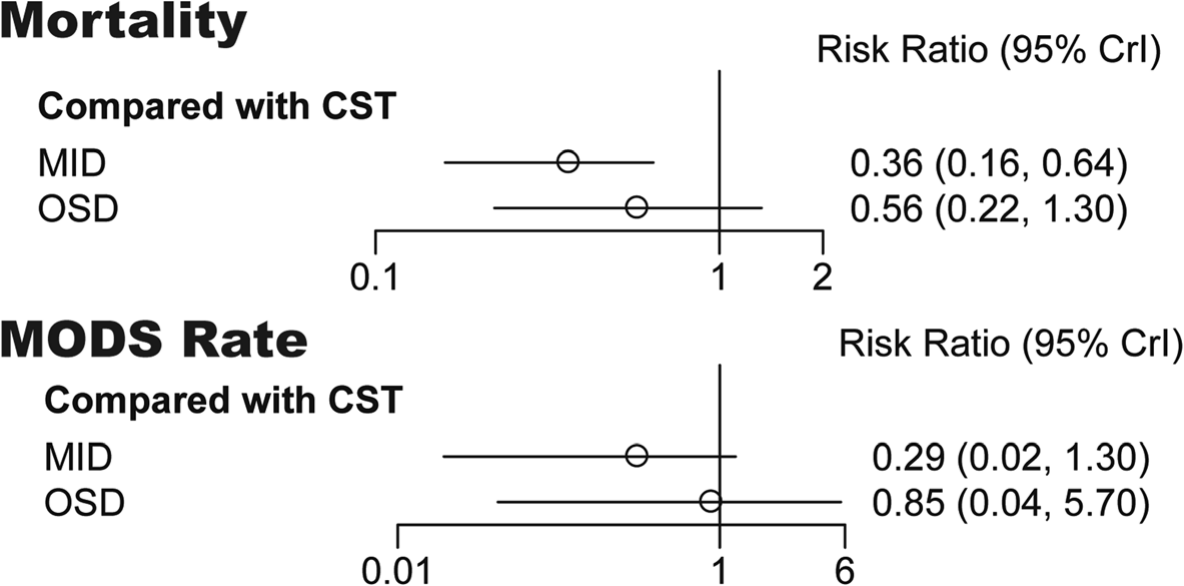
Forest plot for CST, MID and OSD

All these twelve studies contain the data onto mortality (113/931) and six studies for MODS rate (65/406). Comparison of mortality (34/453 in MID and 29/136 in OSD with 50/342 in CST) and MODS rate (15/196 in MID and 33/91 in OSD with 17/119 in CST) of the patients showed that MID could significantly decrease the mortality (pair−wise RR: 0.50, 95%CI 0.21–0.81, I^2^ = 19.9% and network RR: 0.36, 95%CrI 0.16–0.64, I^2^ = 24.1%) but not MODS rate (Fig. 4, Table 2).

**Table 2:** Pair-wise and network estimates of the effects of different treatments compared with CST on mortality and MODS rate

	Comparisons	Risk Ratio (95% CI)		Risk Ratio (95% CrI)		*P* for inconsistency^a^
		Pair-wise meta-analysis	I^2^	Network meta-analysis	I^2^	
Mortality						
Comparison 1						
MID	6	0.50 (0.21, 0.81)	19.9%	0.36 (0.16, 0.64)	24.1%	–
OSD	1	0.95 (0.60, 1.52)	0.0%	0.56 (0.22, 1.30)	0.0%	0.605
Comparison 2						
EMID	4	0.53 (0.32, 0.88)	0.0%	0.43 (0.21, 0.78)	0.0%	0.598
LMID	2	0.25 (0.03, 2.22)	66.2%	0.19 (0.06, 0.47)	24.4%	0.842
EOSD	0	–	–	0.55 (0.12, 2.50)	–	–
LOSD	1	0.57 (0.11, 2.87)	0.0%	0.43 (0.16, 1.10)	0.0%	0.879
Comparison 3						
ESD	2	0.37 (0.10, 1.34)	0.0%	0.24 (0.02, 2.90)	0.0%	–
PCD	2	0.28 (0.02, 3.98)	86.6%	0.23 (0.01, 1.80)	86.8%	–
MIS	2	0.48 (0.24, 0.93)	0.0%	0.42 (0.04, 4.20)	0.0%	–
MODS rate						
Comparison 1						
MID	2	0.40 (0.08, 1.98)	78.4%	0.29 (0.02, 1.30)	76.7%	–
OSD	0	–	–	0.85 (0.04, 5.70)	–	–
Comparison 2						
EMID	1	0.62 (0.26, 1.48)	0.0%	0.43 (0.20, 0.76)	0.0%	–
LMID	1	Can not be calculated	–	0.18 (0.06,0.46)	–	–
EOSD	0	–	–	0.57 (0.11, 2.70)	–	–
LOSD	0	–	–	0.44 (0.16, 1.10)	–	–
Comparison 3						
ESD	0	–		–		–
PCD	1	Can not be calculated	–	3.70^−12^ (9.70^−32^, 0.06)	–	–
MIS	1	0.62 (0.26, 1.48)	0.0%	0.56 (0.03, 1.10)	0.0%	–

^a^ Node-splitting analysis of inconsistency

### Comparison 2-EMID VS. LMID VS. EOSD VS. LOSD VS. CST

**Fig. 5:**
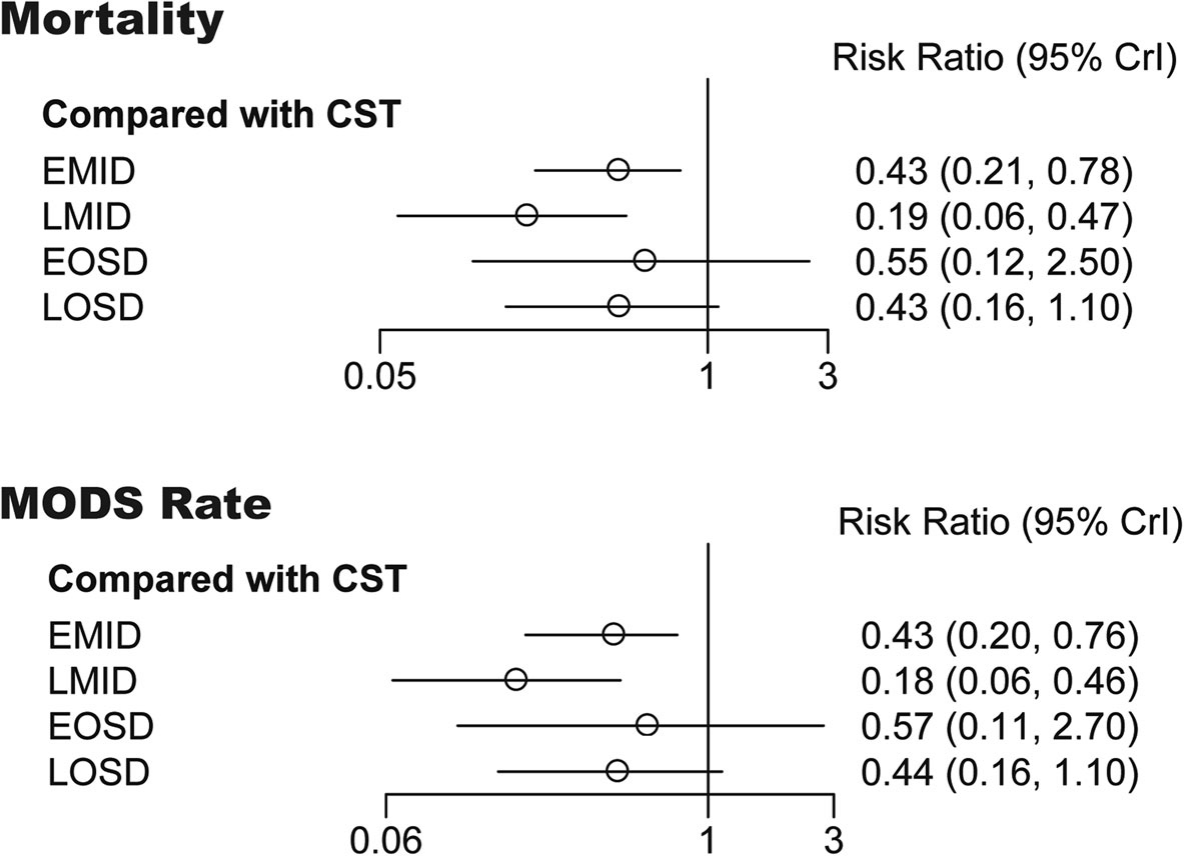
Forest plot for CST, Early MID, MID, Early OSD and OSD

Thirteen studies were taken in this part. Data for direct comparisons and network estimates for both mortality and for MODS rate of SAP/MSAP treated with various ways are shown in the Table 2. We ranked the comparative effects of all treatment against CST. Overall, both early MID and MID can significantly decrease the mortality (RR: 0.43, 95%CrI 0.21–0.78, I^2^ = 0.0% and RR: 0.19, 95%CrI 0.06–0.47, I^2^ = 66.2%) and MODS rate compared to CST (RR: 0.43, 95%CrI 0.20–0.76, I^2^ = 0.0% and RR: 0.18, 95%CrI 0.06–0.46, I^2^ was incalculable), but early PSD or PSD can’t in network estimates (Fig. 5).

### Comparison 3-ESD VS. PCD VS. MIS VS. CST

**Fig. 6:**
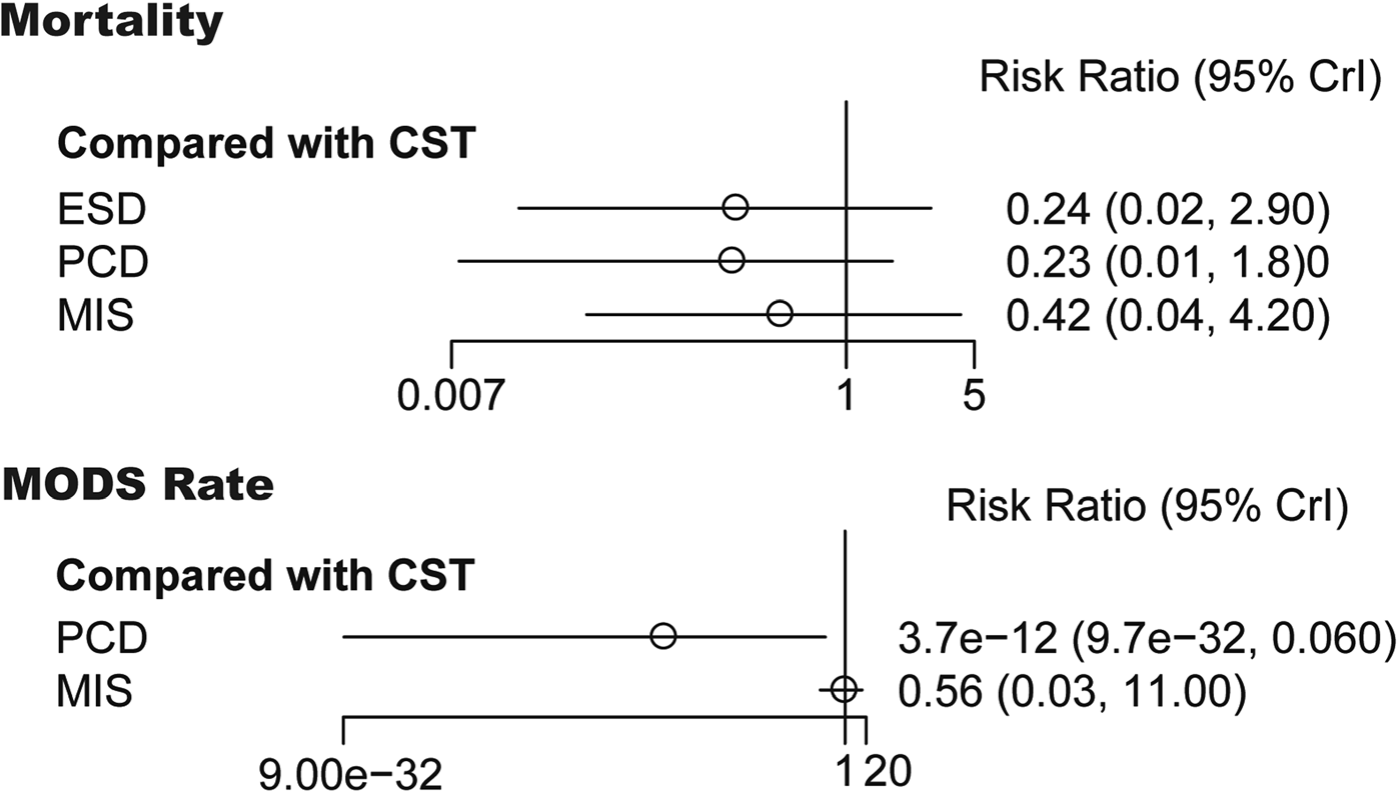
Forest plot for CST, ESD, PCD and MIS

The above result showed the positive effect of MID SAP/MSAP treatment. Then, we proceeded the direct comparisons and network estimates to estimate the mortality and MODS rate of SAP/MSAP treated with various types of MID. Only one direct comparison showed that MIS could significantly decrease the mortality (RR: 0.48, 95%CI 0.24–0.93, I^2^ = 0.0%). The networks of eligible comparisons showed that ESD, PCD and MIS can decrease the mortality but not statistically significant (Fig. 6).

## Discussion

This meta-analysis based on thirteen comparative studies of MID (ESD, PCD, and MIS), OSD and CST, enrolling 931 patients with SAP/MSAP. The results of 453 patients in the MID group, 136 patients in the OSD group and 342 patients in CST group documents the feasibility and potential benefits of MID. No matter early MID and MID could decrease the mortality and MODS rate comparing to CST. Moreover, MID in the later period may be better. OSD had no influence on overall morbidity and MODS rate compared to CST. Several Studies got the same conclusion and suggested that one or more types of MID should be prefered [21].

MID contained several types, such as ESD, PCD and MIS. Despite high popularity of minimally invasive surgery in other surgical disciplines, MID has still not become the gold standard in the treatment for SAP/MSAP [22, 23]. While performing this meta-analysis, we found few randomized controlled trials on this topic, but the sample size is not large enough. As we know, there are several previously published meta-analysis comparing the MID with open approach [24–26]. However, in most of them, there is a methodological bias due to the fact that they were including not only RCT but also some retrospective study in their analysis studies. Therefore, in our analysis, we included only studies reporting as RCT, thus, limiting bias associated with uncertain data.

The mainstream principle of interventions in SAP/MSAP now is that no single approach is appropriate for all patients. The best approach which is adaptable to the individual patient is multimodal. Integrated management of these patients by specialists is essential to achieve a better outcome. The step-up approach involves percutaneous or endoscopic transmural drainage for sepsis control followed by minimally invasive or open necrosectomy as indicated and was recommended in some guidelines [27, 28]. A multi-center RCT showed the result that patients with (suspected) infected necrosis treated with a step-up approach of PCD, followed, if needed, by minimally invasive necrosectomy could be superior to those treated with primary open necrosectomy [15]. Another multicenter RCT designed by the same team suggested that endoscopic transluminal necrosectomy may decrease the risk compared to surgical necrosectomy in some terms, such as new onset multiple organ failure and overall complications [21]. In another study by Sunil Shenvi et al., a randomized controlled trial was designed to establish the benefits of group A (step-up approach as a bridge to surgery) or group B (step-up approach with intention to avoid surgery). The trial was stopped after the first 8 patients randomized into two groups because of difficulty in accrual and poor progress [8].

In this meta-analysis, SAP/MSAP treated with MID would get better prognosis than those treated with CST or OSD. And early MID may achieve better effect than late. But, in this study, there are some aspects which should be improved. The first and the most important limitation is that it is not an interventional study in which improvement could be made. Secondly, the articles included in these studies contain more Chinese ones than English (seven Chinese articles and six English articles). And even in some English articles, the first authors and data were from China. Fortunately, the conclusion of the studies both from Chinese or English articles are the same (Additional file 1: Figure S1 and Additional file 2: Figure S2) and the results of meta-regression suggested language was not statistically significant in the mortality comparison of CST and MID, and CST and EMID (Additional file 3: Table S1). The results showed that MID would get better prognosis. Thirdly, it was hard to identify what is early and what is late. We could only define that treatments do immediately or early reflected in the studies as early. And those treated late or timing not clear in the studies were categorize as late. After that, the timing effect would be weakened. All these above factors can affect the credibility of our meta-analysis.

## Conclusions

The existing evidence of this study shows that the use of MID in SAP/MSAP is reliable and it could be used as early treatment. OSD, if necessary, should be avoided or delayed as long as possible.

## Additional files

Additional file 1: **Figure S1.** Forest plot of Mortality for CST, MID and OSD in Chinese Articles. (JPG 27 kb)

Additional file 2: **Figure S2.** Forest plot of Mortality for CST, MID and OSD in English Articles. (JPG 27 kb)

Additional file 3: **Table S1.** The assessment of heterogeneity by meta-regression. (DOCX 11 kb)

## Data Availability

All data in our study are available from the corresponding authors upon reasonable request.

## Abbreviations

AP: Acute pancreatitis
CST: Conservative treatment
EMID: Early minimally invasive drainage
EOSD: Early open surgical debridement
ESD: Endoscopic drainage
LMID: Late minimally invasive drainage
LOSD: Late open surgical debridement
MID: Minimally invasive drainage
MIS: Minimally invasive surgery
MODS: Multiple organ dysfunction syndrome
MSAP: Moderately severe acute pancreatitis
OSD: Open surgical debridement
PCD: Percutaneous catheter drainage
RCT: Randomized controlled trial
RR: Risk ratio
SAP: Severe acute pancreatitis
