# Supplementary figures and images for: Minimally invasive drainage versus open surgical debridement in SAP/SMAP – a network meta-analysis
Source: BMC Gastroenterol. 2019 Oct 21;19:168. doi: 10.1186/s12876-019-1078-x (PMC6802312; doi:10.1186/s12876-019-1078-x)

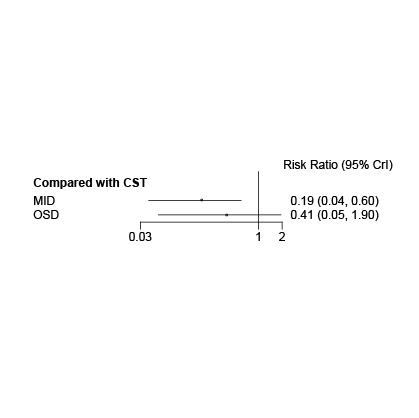

Supplement: Supplementary file 1 — Figure S1. Forest plot of Mortality for CST, MID and OSD in Chinese Articles. (JPG 27 kb) [file 12876_2019_1078_MOESM1_ESM.jpg]

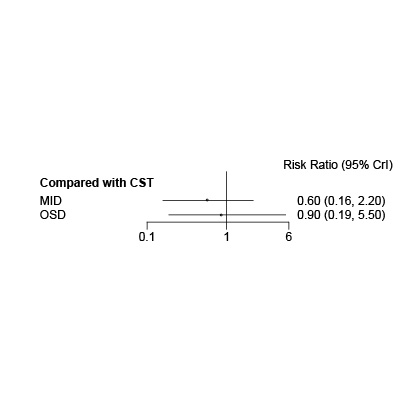

Supplement: Supplementary file 2 — Figure S2. Forest plot of Mortality for CST, MID and OSD in English Articles. (JPG 27 kb) [file 12876_2019_1078_MOESM2_ESM.jpg]
