# Supplementary material for: Minimally invasive drainage versus open surgical debridement in SAP/SMAP – a network meta-analysis
Source: BMC Gastroenterol. 2019 Oct 21;19:168. doi: 10.1186/s12876-019-1078-x (PMC6802312; doi:10.1186/s12876-019-1078-x)
Supplement: Supplementary file 3 — Table S1. The assessment of heterogeneity by meta-regression. (DOCX 11 kb) [file 12876_2019_1078_MOESM3_ESM.docx]

Table S1. The assessment of heterogeneity by meta-regression

|  | CST and MID mortality | |  | CST and EMID mortality | |
| --- | --- | --- | --- | --- | --- |
|  | estimate(95%CI) | p |  | estimate(95%CI) | p |
| Year | -0.103(-0.390,0.185) | 0.4845 |  | -0.098(-0.394,0.197) | 0.5146 |
| Language(English) | 3.267(-0.807,7.340) | 0.116 |  | 1.243(-0.764,3.250) | 0.2247 |
| Sample Size(>100 cases) | -1.9363(-4.959,1.087) | 0.2093 |  | -- | -- |
